# Quarantine Stressing Voluntary Compliance

**DOI:** 10.3201/eid1111.050661

**Published:** 2005-11

**Authors:** Cleto DiGiovanni, Nancy Bowen, Michele Ginsberg, Gregory Giles

**Affiliations:** * Defense Threat Reduction Agency, Fort Belvoir, Virginia, USA; † County of San Diego Health and Human Services Agency, San Diego, California, USA; ‡ Hicks & Associates, Inc., Arlington, Virginia, USA

**Keywords:** quarantine (human), bioterrorism, infection control, risk communications, consequence management, table-top exercises, dispatch

## Abstract

A 1-day table-top exercise in San Diego, California, in December 2004 emphasized voluntary compliance with home quarantine to control an emerging infectious disease outbreak. The exercise heightened local civilian-military collaboration in public health emergency management. Addressing concerns about lost income by residents in quarantine was particularly challenging.

In a study of quarantine implementation in the greater Toronto area during its 2003 outbreak of severe acute respiratory syndrome (SARS) (*1*), investigators identified several factors that influenced compliance with quarantine restrictions (with rare exceptions, identified contacts of SARS patients in the area were quarantined in their own homes). Key factors were fear of income lost while quarantined, consistent information about the threat and measures to contain it, and adequate logistic and psychosocial support to those confined. Using these findings, we organized a 1-day table-top exercise in San Diego, California, in late 2004 that revolved around an emerging infectious disease caused by an easily transmitted novel respiratory virus.

## The Study

Our twin objectives were to promote coordinated implementation of quarantine measures by the several local military and civilian agencies and jurisdictions in San Diego County and to emphasize initial decisions that encouraged voluntary compliance. In this exercise, quarantine referred to restriction at home of possible carriers of the agent, who were identified through contact tracing. We also challenged local military commanders to preserve operational readiness as scenario events unfolded.

Before this exercise, quarantine plans in San Diego County were in their formative stages. We did not, therefore, organize a conventional table-top exercise that tested an existing plan and identified its gaps. Instead, we used the exercise as a stimulant for further developing that plan; throughout the several months that preceded the exercise, we worked closely with participants to ensure that the exercise achieved their objectives and goals.

Contact tracing, issuing verbal quarantine orders and instructions, and delivering news to the public were generally well coordinated. When concerns about privacy issues arose in framing public announcements, a US Department of Health and Human Services representative assured participants that Health Insurance Portability and Accountability Act Privacy Rule provisions would not restrict release of protected health information, as necessary, to inform the public of the crisis and instruct them about protective measures.

The region's military public affairs officers formed a joint information bureau to prepare press releases and sent a representative to the county's emergency operations center to coordinate military-civilian announcements to the public. Civilian and military mental health providers, however, lacked coordination in the delivery of their services.

County officials established a phone bank with a single 800-number for the use of all county residents (military and civilian), staffed it with operators from several agencies, and provided the operators with Internet-based training to promote consistency in information released to the public. Service organizations provided logistic support to those in home quarantine.

Military commanders pledged cooperation with civilian public health and emergency operations center officials but were unable to commit resources in advance because they needed to protect their operational readiness. The San Diego County superintendent of schools, who coordinates all public schools in the county, including those on military bases, organized Web- and television-based distance-learning programs so that quarantined students could continue to receive instruction and submit assignments.

Civilian law enforcement officials characterized the public health crisis in the exercise scenario as "unprecedented." They also frequently expressed concerns about carrying out enforcement measures that were requested by civilian public health authorities and urged county officials to emphasize public education to minimize the need for enforcement.

When members of the community who were ordered into quarantine raised questions about payment of their wages and salaries while away from their jobs, answers varied. Military personnel were assured of continuing income, but some government employees and the privately employed or self-employed received no assurances. This issue remained unresolved throughout the exercise because no program or funds to compensate for income lost by still-healthy contacts while in quarantine existed at the local, state, or federal level. In the absence of income insurance, officials could only appeal to the public's sense of civic responsibility to comply with quarantine restrictions.

Border health officials agreed that health warnings could be posted, and perhaps health-related information provided, at the county's border crossings with Mexico, but any general increase in intensity of health screenings there that lengthened processing of border crossers even minimally would impede the flow of traffic and become untenable. (The San Ysidro border crossing, between San Diego County and Mexico, is the busiest US port of entry. Future construction of new traffic lanes may allow more time for health screenings, when necessary.) Making border health screenings even more problematic at the time of this exercise was the shortage of both federal and county personnel to carry them out.

## Conclusions

Several key lessons were identified for future exercise planning. First, memoranda of agreement (MOA) or understanding (MOU) should be developed. Although San Diego County has had a variety of real-world disasters, it had relatively little experience with an evolving public health crisis as depicted in the scenario. Thus, forming collaborative and coordinated incident command among local civilian and military officials took some effort. The lack of either formal collaborative agreements or even informal collaborative relationships among local civilian and local military commanders in the overall management of a declared communitywide public health emergency was highlighted by this exercise. In areas with multiple jurisdictions, whether civilian, military, federal, or tribal, planning for quarantine must be based on collaborative command and control, best articulated in MOA or MOU.

Second, early collaboration of nonmedical officials in exercise planning should be obtained. Quarantine measures are initiated by health officers, but they are largely implemented by nonmedical personnel. Success of our exercise required active collaboration by a wide variety of local public officials and nonmedical military officers; some required convincing that they had crucial roles in what they initially thought was a purely medical exercise. Communities planning to exercise quarantine in collaboration with local military commanders should enlist, early in their planning, the cooperation of local military force protection as well as medical corps officers. Private sector participation in this exercise was valuable. Implementing quarantine in a community will have an immediate impact on the business sector, and its cooperation will promote compliance. Administrative, nursing, and medical representatives from the private hospitals in the community also need to participate and develop consistent approaches to human resources issues, such as pay and benefits for quarantined employees, including those in work quarantine, i.e., those who may have to leave their homes and return to work because of critical staff shortages during an epidemic.

A third key lesson is to include likely challenges, even sensitive ones without current solutions. This exercise did not resolve the critical issue of income protection for those in quarantine but did identify the lack of this protection as a potential impediment to voluntary compliance with quarantine, highlight its importance for further discussion, and force participants to craft appeals to the residents' sense of civic responsibility to mitigate concerns of income loss.

A fourth lesson is to use exercises to help develop quarantine plans, not only to test them. Web-based guidance by the federal government to civilian public health authorities (*2*) and Department of Defense directives to military commanders (*3*) will encourage quarantine planning. Staging an exercise will develop this capacity and pinpoint deficiencies in interagency collaboration.
